# Report of Discussions at the Ohio State Dental Association

**Published:** 1868-12

**Authors:** 


### REPORT OF DISCUSSIONS AT THE OHIO STATE DENTAL ASSOCIATION, DEC. 1ST AND 2D.

REPORTED BY ———

*First Subject* — THE MEANS OF CONTROLLING THE FLOW OF SALIVA DURING OPERATIONS.

Dr. BUTLER: I suppose it is well understood by all that one great difficulty we have to contend with in filling teeth is the saliva, or fluids of the mouth, and the great difficulty in controlling them so that we may be able to make successful operations, especially upon the inferior teeth. Of course men have their peculiar modes of doing this, and stick to them with a great deal of tenacity. There are the rubber dam, the napkin, the duct compressor, &c.

Some will condemn the use of one mode for all cases. I have tried nearly all the different appliances that have been brought before the profession, and, notwithstanding some claim to the contrary, there are some cases that I am unable, with any thing that I have seen, to control the saliva so that I can make such fillings as I would desire.

With these general statements, I would simply say that I do not think that any one mode is the best in all cases. I have not had much practical experience in the rubber dam, but would say that in many cases it seems to be the only thing that will protect the case so that you may operate upon the tooth. The greatest objection I have to it is that it requires considerable ingenuity and skill to use it, in such a manner as to answer the purpose for which it is designed.

Unless the preparation is made of a good article, and made nicely, it is liable to tear. There is also a difficulty in turning the edge down, which is the great point. After it has passed over the tooth to be operated upon, it must be turned down and the silk thread tied above it, so that it will not slip. In my own practice I resort to the use of small napkins, and believe that the less complicated the appliances for this purpose, the better. There is a sort of repugnance to putting any of these claptraps in the mouth. They seem to excite the flow of saliva, and you actually defeat the attempt to control the moisture.

The use of large napkins, I think, is inadmissible. They are in the way of the operator, and fill the mouth so much, and annoy the patient so, that they do more harm than good, and then you can not change them so readily as small ones. There are a great many operations we are called upon to make now-a-days, which it is impossible to complete without changing the napkin several times. The manner of introducing and consolidating the gold requires time, and the cavity must be free from moisture.

In the first place, to give a little detail of the procedure: In proximal cavities, I would use the wedge of wood, putting it in each side of the tooth I am going to work upon; that shuts off any flow of moisture at the base of the cavity and gives a view of the lower margin, and protects the gum in the operation of packing the gold and finishing the filling. If there was only that one advantage in the wedge, that is sufficient to balance all the inconvenience of its introduction. In those cases where the teeth are a little feeble and loose, and where there is that spongy condition of the gums, where it is difficult to suppress the flow of moisture, I find by wedging up several teeth along, you succeed quite well in shutting off the moisture, which is so great an annoyance.

Then in using napkins, I would use no others than those that can be readily folded in a convenient shape to place under the tongue and outside the labial surface, using smaller ones outside than inside, so the tongue will not be forced back in swallowing. There are some patients who are not able, or do not know how to swallow if there is any thing in the mouth, and with them it will be difficult to control the moisture. By placing the napkin in on the side where you are going to work, and allowing it to come round perhaps two-thirds of the way, it may be easily changed when it becomes saturated. But this is a matter that very few seem to have become master of—just handling those little compresses so as to accomplish the object.

I would simply state in addition to the use of these small napkins—going back to the rubber dam: Cases I can not control with the napkins, I put in the rubber dam, and in some cases I believe it is necessary. This flow of moisture must be controlled, though it demands time, if we would have the operation such as we would expect to get good results from. There are many I know that come short of obtaining the best results, from the fact of being unable to control the moisture. You may talk about submarine filling, but I would not resort to it if it could be avoided. That is the last resort, and then only an imperfect operation can be made. The large gold fillings that are demanded at our hands, require different manipulations throughout from those of ten or fifteen years ago. There are a great many fillings now made that ten years ago would have been thought impossible, especially in the inferior teeth. There is not much difficulty in controlling the moisture on the superior teeth and making operations, but it is with the inferior that the great difficulty occurs.

Dr. Spellman: I am constantly using the rubber dam, but it is often very annoying. Sometimes I am on the eve of giving it up, because it slips off when I get it placed. When the rubber dam is properly adjusted, you can draw the mouth up as you wish, and go into the reception room and chat with patients, though the filling is not completed. When properly put on it is impossible for the water to pass between that and the tooth. In filling the inferior teeth, I regard it an indispensable article. When operating on a bicuspid I very often, in order to succeed with the rubber dam, place it over three teeth and operate on the middle one. It is almost impossible to put the rubber over the inferior cuspid in such a way as that the water will not leak through. In such cases I always have cut up, in readiness, pieces of spunk, and if it leaks and they become wet, I change them as Dr. Butler changes his napkins. You will see at once when the spunk is saturated and should be changed. With regard to the inferior incisors, it is very difficult to apply the rubber dam, yet it can be done by taking a piece of silver wire, or if you have not that, a very small wrapping wire, and, bringing forward, hold it with a blunt instrument and crowd it below the crown of the tooth; then pass your rubber down as near the wire as possible; then slipping the silk over this twice after, and with a fine instrument slip the edge of it over the wire. It is so liable to slip up, but with the wire it can be held down. So far as the upper teeth are concerned, I use it a great deal, though I don't regard it as indispensable any where, except for the inferior teeth. In filling the superior bicuspids the wedge is my dependence. But we find that the gums secrete a kind of watery mucus that is not naturally a product of the saliva, by glands, and when engaged in an operation of filling, you will discover it is becoming moist. This spunk, if pressed into the interstices between the teeth, in such cases, will always show you when it is time to change it. When there is danger of water I never fill approximate cavities, or attempt to do it, without pressing into the interstices between the teeth, pieces of spunk, in this way, and if the wedge passes far enough above the base of the cavity, so as to put a very thin piece of spunk in, I prefer it, for, although you dry off the wood well, the water will work through it and the first pellets will become a little dampened. I would rather have every point clean and dry, for when you come to filling the upper margin you can not polish or finish off with so nice and smooth a surface as if it had not got wet or dampened by contact with the wood. I use bibulous paper, where Dr. Butler uses napkins ; I don't know whether it is better or as good. [A member, "It is good, though expensive."] I use it because I thought it was not so expensive. A quire of this paper will last a good while. It is rather a delicate tissue paper, and not so rough to the mouth as supposed. It will contain a great deal of water. Formerly, in using it, I took and folded it closely, one layer over another, folding it compactly and rendering it hard, supposing that the more I got in the more water it would contain. The paper is capable of great expansion, and will contain water in proportion as you allow it to expand. Afterwards I found that half the amount of paper would hold as much water and, folded more loosely, it was not so harsh or offensive to the soft, velvet-like tissue it is laid upon.

Dr. BUFFETT: I can not tell you how to control the flow of saliva. I can tell how I *attempt* to do it. I confess I have not received very much benefit from the rubber dam. It has been a failure, in my hands, to a great extent. I use the holder sometimes for holding the cheek back, and in cases which I consider difficult, as the inferior teeth, I sometimes have the strap to pass around the neck and head, and held by the patient. That I consider an indispensable appliance, and on the inferior teeth, I depend almost entirely on napkins, and, occasionally, on the tongue-holder. Instead of the napkin, I use linen cloth, called diaper, containing considerable starch, so as to contain a certain amount of stiffness ; if it is washed it is sometimes too flimsy. I cut it into pieces from an inch to four inches square, placing these small pieces and pressing them under the tongue, and the large napkin under the end folded in a strip. You can change either the larger or smaller napkins as many times as you wish.

The fewer things put in the mouth the better we can operate, and with more ease to ourselves and patients. It depends a great deal on the firmness, as you may say, of the operator. If you determine to control the flow of saliva and let the patient understand what you intend, you will be more likely to succeed. But if you go at it indifferently and undecided, your patient will think there is going to be a great deal of trouble, and they will get excited so that you can not make a good operation. Even if I think there is danger I don't tell them. If I fail I try again.

Dr. HERRIOTT said he tried to control the condition of the patient. He applied something to the teeth after they were excavated—applied white wax and sealed up the cavity, and delayed the filling a day or two. Had this engagement arranged beforehand. Then when the patient returned there was no excited condition of the patient, as immediately after the excavation of the teeth, when the flow of saliva would be greater than if the patient had been quiet for a day or two.

Dr. N. W. WILLIAMS, for the last few weeks, had been using the new duct compressor (Smith's). The part that passes under the chin has a lateral motion, also, that which passes inside. His way of using it is to cut out a piece of spunk, something in the shape of a half moon, and place on the ducts, and then a small napkin laid round inside of the teeth, under the tongue, placing the duct compressor on that and pressing down as tightly as it would admit of without irritating the muscles. He had succeeded better in controlling the flow of saliva with that apparatus than any thing he has tried before. Before he got that he had succeeded pretty well by the use of the napkin and spunk. He had, by directing the patient to crook the finger, and place the napkin in the mouth and hold it, succeeded in cases where he had failed before. He thought spunk controlled the flow of saliva better even than the use of napkins, for, as Dr. Spellman says, we can, by using it, generally tell when there is approaching danger. He had often succeeded by having a piece of spunk near at hand and applying it. But by applying the spunk under the tongue, over the saliva ducts, he had found particular advantage.

### *Second Subject*—WHAT ARE THE INDICATIONS FOR THE EXTRACTION OF TEETH.

Dr. DECAMP said he was aware that there is a great variety of opinions with regard to the indications for the extraction of teeth. Some would regard the fact of the pulp of a tooth being exposed, simply as an indication for extraction, and perhaps a majority of practicing Dentists go upon the principle that where the pulp of the tooth is actually exposed and aching, the only remedy or the only course with them is to remove it, whereas in point of fact with the more skillful in the profession this is not an indication at all for extraction. With others, where they find the tooth diseased at the root, or the pulp already dead and alveolar abscess formed, or gum boils as they are called, or some periostial irritation, they would regard these conditions, indications for the extraction of teeth. There are others again, that would not regard these as indicating extraction. Hence, I am aware that on this subject there would be a great variety of opinions. The time is approaching when neither of these indications will be regarded by any respectable man in the profession as an indication for the extraction of teeth.

Then, "What are the indications for the extraction of teeth?" is an important question. It is not only important to the profession, but certainly more important to the patients, to every one who has diseased or decayed teeth.

This subject will never be sufficiently appreciated until we all learn, both people and practitioners, the value of the teeth as they ought to be valued. And perhaps no tooth should be extracted from either of the indications named, tha is, from exposed pulp, or decay, disease, alveolar abscess, without efforts to preserve it.

In my judgment the true policy would be to extract no teeth in any case but as a last resort, particularly so with children's teeth, if they become diseased before the eruption of the second teeth or second dentition.

Dr. BUTLER: I have no special theory to offer at this time. The indications for the extraction of teeth are but few, if we had the control of all the circumstances; or rather, of all the cases that present themselves, I doubt whether many, if any would willingly consent to such an operation unless it were as a last resort, if the circumstances would permit of any other operation. And that term circumstances will apply in various directions. Pecuniarily there are very many that can not afford to have anything else done, and can illy afford that; but there is no one that is sufficiently insensible to endure the pain of these offending organs. Notwithstanding all that, there are those extracted that ought not to be, and that could be very much better treated.

I will confine myself in what little I have to say, to one particular branch; that is, to the *teeth of children* You know that there is a very great diversity of opinion even on that subject—the extraction of the deciduous teeth. The root passing through the gum, causing ulceration or fistulous openings as we frequently see. In such cases he thought they were justified in extracting them on account of the irritation. As a general thing persons should be very cautious about extracting temporary teeth prematurely. He had the other day a case at his office in which the inferior lateral incisor was firm and no absorption was induced except on the side of the incoming tooth. The tooth had taken a wrong direction and came up back of the roots of the incisor, that of course was an indication for extraction.

With regard to the six year old molars, he always contended that these teeth should remain in order to have a proper contour of the mouth. Every proper means should be taken to preserve them. It is a severe test for children ten or twelve years of age to have teeth extracted, it prejudices them forever after against going to a Dental office.

If he were to sacrifice a six year old molar on one side, he would be very much opposed to extracting the one on the consider it a wholesale slaughter of the teeth. He thought opposite side, if it was not badly decayed, because he would this poor policy; on the same principle if we extracted a superior bicuspid on one side we might extract the one on the opposite side, because it might throw the teeth out of the mesial line of the arch. If that was likely to take place we could by mechanical appliances throw it over to its proper place. He would be very far from extracting a tooth opposite another which it had been necessary to extract.

He desired to make an explanation, so that what he had said should not be construed as a wholesale slaughter of the teeth. He had prefaced what he had said on this very strongly, and thought they would find no one go more strenuously against wholesale removal of teeth than himself. But if you find one of the six year old molars decayed so as to be necessary to extract it, we find in a majority of cases, a very great majority, perhaps in nine hundred and ninety-nine cases in a thousand that the opposite one is decayed; indications to my mind are simple. When they interfere with the incoming teeth or tooth, or there is some diseased condition so that they can no longer be tolerated they may be removed. And that is all that I recognize as indications for their removal. Leaving the deciduous teeth with what I have said in relation to them, and passing to the six-year old molars, I will notice the indications to my mind for their removal—and I may differ from many. I would retain them, if possible, until the full development of the arch, which depends so much on them. If they must be sacrificed in time, I would endeavor to retain them as long as possible. But there may be such a degree of irritation kept up that the indication is imperative that they should be removed at once. We should be governed, at least, to a great extent in such operations by the want of space or room in the arch. We should not remove them in those cases if there is any possible way to retain them. Keep them and let them act as fulcrums as long as may be, so that the arch may be expanded.

But if they must be removed, I would rather take out two than one, so as not to have a one-sided denture. We find as it is often said, very few straight faces with the nose in the middle of the face, or the teeth standing so that it gives you a straight denture. The mesial line of the teeth stands to one side of the mesial line of the face. And that would be the result in a great majority of cases where one is removed. So if I should be obliged to take out one, I would try to get a good excuse to take one out of the opposite side, so as to have straight work.

Dr. Buffett: the rule for extracting teeth should be, whenever we can save a tooth and make it more useful than an artificial tooth, do so. If we have not the skill to save the tooth, and another has, we should refer it to him. We are not justified in removing a tooth which would be more useful than an artificial tooth. In regard to extracting the teeth of children, I do not think we need extract one in five hundred cases presented to us, unless it be the removal of a root. A majority of them can be tolerated in the child's mouth even if ulceration has taken place. They can be put in a healthy condition, and the child have a well developed set of teeth.

As an illustration, a case came under my notice not a great while ago, where the lateral (?) had taken the place of the permanent cuspid, and the cuspid had taken the place of the permanent lateral. (?) There had been a perfect transposition if I may use the term, merely by extracting the temporary tooth two years before it should have been. As to the extraction of the six year old molars, I think they should be kept in until the arch is well formed. There are some who will not tolerate a tooth with a nerve destroyed, for instance, scrofulous persons; but these are exceptions.

In extracting permanent six year old molars for artificial teeth, it is often a question how many should remain. Where part are gone and others can be saved, it is a question whether to leave in two, three or four. Some advocate the plan of leaving only one. It is a question whether we should leave sometimes the cuspid teeth that we find sound. Where the molars, bicuspids and incisors are gone, and the cuspids in good condition, I have always left them.

Dr. Dunlap thought he should be very sorry, especially in healthy cases, to extract them. It was a new idea to him, he did not know but that it would be a good idea in order to keep a straight denture. He was very much interested in the discussion, and would like to hear from others on this subject.

Dr. Kelsey said, one indication for the extraction of teeth is when there is too much irritation, and the teeth are past filling they should be removed even if it required wholesale extraction. With regard to the temporary teeth he thought too many extracted them prematurely. He thought they ought to remain unless the other teeth had worked their way out either anteriorly or posteriorly, or were thrown out also, so that if it becomes necessary to extract one, the indications are pretty certain that the other will have to be sacrificed also. It was said our mechanical or artificial appliances can come in and mend it up. There is too much mending up, and a mighty poor mending it is. A great deal of mending might be saved. By the sacrifice of one or two teeth we might save all the rest on that side, whether superior or inferior. In the attempt to save the two you may lose four or even more.

He wished to express no feeling, but desired only to express his ideas. He knew there was a tendency in the teeth of both sides to decay. Yet we had seen teeth stand undecayed on one side while on the opposite side they were decayed very much. In many instances where there was the first indication of decay, the patient had come and availed himself of the advantages of the profession and had the tooth filled in time, and had it preserved for years; whereas the other, perhaps, had gone on for years without the knowledge of the patient, until it had decayed so much as to be past remedy.

Dr. HORTON said he did not intend to make any remarks. He saw gentlemen around him of more experience, and who ought to be better prepared to make remarks on subjects of this kind. But he was willing as one of the profession had said, "to let the darkness out of him." Whether what he said agreed with what had been expressed or not, if the darkness were let out of him the light might get in.

The indication for the extraction of teeth are various. He did not think that any one could give specific rules by which all practitioners could be governed, because all do not see through the same eyes, and all do not have the same classes of patients to treat.

If we were left to our better judgment we would not always pursue the plans we do, but we are surrounded by circumstances which prevents us from being sole actors in the drama. There are those who come to us who have not the money to pay or the time to spend to have their teeth properly treated, and they demand an operation which in our better judgment we would not perform if the patient were otherwise situated.

Here comes a servant girl for instance—he occasionally had one of that kind—whose time was occupied at manual labor in the kitchen for a large family. She finds it difficult to leave the house and be away long and perhaps not oftener than once a week, while we would require her presence to make a successful job, three times a week at least, and perhaps for two or three consecutive days. But she having sold herself as far as her time is concerned, imperatively demands the performance of an operation which our better judgments would otherwise condemn, but which our benevolence might under the circumstances prompt us to do. He had thus sometimes extracted teeth when the indications were that with the proper treatment he could have saved them; but the fact was, the patient then and there demanded them to be extracted. Although it went against his conscience in some of these cases to do so, yet he knew he was relieving them from a burdensome pain, and enabled them to go on and perform their duties.

He held that the absorption of the roots of deciduous teeth is a vital operation. In many children, and especially in Cleveland, for some cause not necessary to explain the children had very bad teeth, especially among what are called the better classes. There was scarcely a week that he had not such cases to treat. In many cases the crown of the tooth was all gone and nothing remained but an ugly root in the jaw, which was a source of much trouble. In some children twelve years of age, intense swelling of the face resulted from a little exposure, or on taking cold, inflammation and suppuration followed in their natural course. In almost all such cases extraction of the teeth was the only relief. If palliatives are used it comes to that point before long anyhow.

There are other examples,—here is a case of a gentleman within a day or two, who was suffering from neuralgic pains in the head, face and shoulders; and, in short, the entire man was out of order. His case required and ought to have had an intelligent physician for six months. He came with intense suffering and required the extraction of the tooth. He extracted the gentleman's tooth under protest, and undertook to tell him what would be the ultimate result if he would put himself under the care of an intelligent physician; that he would become bodily well, and that there would be no doubt that this difficulty would entirely subside. He had no patience to do that, and declared he could not endure it another day, and urged the extraction of the tooth. Under circumstances he extracted the tooth, but gave the patient to understand that he did it under protest. The next day he was comparatively well and attended to his business. But that did not remove the cause, for the whole difficulty was in him. The indications there were not strong, they were any thing else but that, but he had come to that point where there seemed to him to be no other remedy. He would not go anywhere else, and would not take the physician's advice or medicine. This to an ordinary observer might have appeared to be an indication for the extraction of the tooth, but to him it was no such indication.

In relation to the removal of the teeth of children where there is a contracted condition or want of expansion of the jaw or maxillary bones; he very frequently hesitated about extracting the teeth, and confessed it was a difficult question sometimes to decide, when parents demanded their extraction, to make room for the incoming process. In most cases where the crowns of the teeth are left, and there is no abcess or local condition injurious to the health, he rarely extracted the teeth the first time he saw the child, but would take time and see the child two or three times. He confessed he was often puzzled about this matter. Parents would rush to him, expecting him to perform that which they demanded, though, perhaps, in nine cases out of ten it may be an injury to the patients. In such cases he took some time to consider, though in a few instances he had yielded and extracted the teeth.

Dr. George W. Keely said if there was any one thing in the profession that made him more furious than another, it was seeing cases and hearing of cases where these good sound organs are slaughtered. He was very much pleased with the remarks of Dr. Buffet in regard to the indications for the extraction of teeth. That was that a tooth should never be extracted, if it is possible to save it and make it better than an artificial one. Dr. Keely thought all would agree that a miserably poor natural tooth was better than a good artificial one; consequently, if they have an opportunity to save a natural tooth, it was their duty to do it. He knew it was too common a thing in the profession to extract sound teeth. Patients would come to them, having an idea, perhaps, that a partial set of teeth would cost as much as a full set, and wanted a full set, as the expense would be the same, and they would insist on having good sound teeth extracted, even molars, where they would antagonize. All in the house knew of cases where such teeth had been extracted. To call such a practice villainy was too mild a term; to call it an outrage was not language sufficiently strong; there was not a single invective in the language strong enough to denounce this act in its proper form. [A member, "That's so."] He would give a case that came under his notice last week. A young lady had come from a Cincinnati Dentist, of whom he thought they had read enough and heard enough. The young lady, by the way, was quite beautiful. She discovered coming out between the bicuspids a tooth having the appearance of an eye tooth. The Dentist had a fair opportunity, and could have regulated it by widening the arch. Instead of that he extracted the cuspids and ruined the appearance of the young lady forever. In a case of that kind the indications to him would have been, if he failed to get the patient's time to regulate the teeth and get room for them, to have taken out the bicuspids. He would rather have sacrificed both bicuspids than the eye teeth. The contour of her face would have been better preserved if both bicuspids had been taken out and the cuspids left.

Another thing. He did not think because a patient comes to us and insists on having a certain tooth extracted, that we are in duty bound to do such a thing. Said he, "If I were not ashamed I would tell you what I once did. I guess I will do it any how. A lady teacher in our school desired me to extract two bicuspids, two molars and three other teeth. She came four or five different times. I told her positively that I would not do it. Finally she came in a fit of desperation. I extracted them. They could have been saved, though they were somewhat decayed. One of the bicuspids was considerably diseased, but I thought could be saved. I extracted those teeth and have not got over it yet, and never expect to. I put them away in a little tin box. And in case I see a person put fillings in a dozen teeth in an hour, and extract others where they are not justified in doing so, I go to my little tin box containing these teeth, which I extracted ten years ago, and say 'these seven teeth you extracted when you ought not.' I go to the glass and say, 'Keely, did you do this?' 'Why no; I didn't!' Yet I can not get out of it—can not deny it. I don't know that any of you have been guilty of any such thing, but you have doubtless seen such things. I don't think money would hire me to do it again." Since then he had been requested to do the same by other patients, and when he refused they would probably reply that they could go elsewhere and get it done. He would tell them "Yes, if they had perfectly sound teeth, they could get them extracted and get a miserable substitute—an outrageous miserable practice."

He thought it important to save every tooth that can possibly be saved. Only week before last he saw a case of a lady whose first and second molars were in pretty good condition and antagonized, who had come to him to get them extracted. He told her that no where could she ever get any teeth as good as those four teeth. She afterward came back to his office with a new set of teeth and said: "Dr. Keely, you would not extract my teeth; see what a fine set of teeth I have now." The indications were not for the extraction of those natural organs, but to preserve them, in a case like that, and put in a partial set.

He spoke of a case of a man within some ten miles of him, that traveled through the country considerably as a

Dentist. He never extracted a tooth without making a thorough diagnosis, as an old physician would. His perceptive powers were remarkable. He would take his buggy and attend to a great number of cases in a single day. In one day he made a diagnosis of eighty five cases, and came to the conclusion that every tooth must be extracted. He extracted them and came home at night very much wearied. We could imagine how many of those teeth might have been saved. Three-fourths of them might probably have been saved to the patients.

Dr. SMITH thought in the matter of extracting teeth we should apply the golden rule, and do to others as we would be done by under similar circumstances; he thought that was the only true test—a test patients has a right to look for when putting themselves in the hands of the practitioner. There was, he thought, two extremes: those who tried to *save all* and those who *took out all.* He was struck with the remarks of Dr. Keely, particularly the case he mentioned of taking out those good teeth. Of course the doctor had now grown older and wiser. We may not be able to decide, or can not pretend to decide, what should be done in certain cases, until the cases are presented to us. He condemned the idea of sacrificing the opposite tooth, where one must be extracted; if there was an anticipation that it would be lost in a short time, he might extract it.

Dr. BERRY had but a word to say. He thought one cause why teeth were sometimes extracted when they should not be, was the want of faith in the patient to save them. A great many who call on a Dentist have no faith and no patience or inclination to be governed by them.

Dr. HERRIOTT desired to know what Dr. Keely meant by a miserably poor tooth.

Dr. KEELY remarked that it would be a tooth partly broken off, half gone and the nerve destroyed, and in order to preserve it the tooth would have to be filled and built up. He would take, as an example, a molar such as he filled yesterday. They could imagine how large the cavity was when he informed them that he put seven sheets of tin foil into it. That he would call a miserably poor tooth, in comparison with a tooth needing an ordinary filling.

Dr. Herriott said he would consider an artificial tooth better than one filled with seven sheets of tin foil.

Dr. Keely replied that this was a first molar, and he would wager his existence that it would be good for ten years. The patient was not able to have it filled with any thing better.

Dr. Harroun said that while the gentlemen were discussing the subject, and especially the cases referred to by Dr. Keely, it occurred to him that there were cases where there might be pretty strong indications for extracting teeth for the sake of educating the patients in the duty of preserving their teeth. He related an instance or two in his own practice, where he thought he had been justified in whipping patients into the discharge of their duty by extracting teeth when they demanded it, when by proper treatment they might have been saved.

Dr. Spellman desired to reply to Dr. Herriott. Dr. Herriott held that an artificial tooth was better than a tooth filled in the manner described by Dr. Keely, in which he inserted seven leaves of tin foil. The contrast between the condition of a person who has artificial teeth, and one who has the natural teeth, is the only phase of the subject to be looked at. When a person has lost one of these organs which God has given her, it can never be returned. It is gone, gone forever. If she don't like it she is compelled to submit to it. If she proposes to replace it, what condition is she in during the rest of her days? If she intends to keep a substitute in its place, she must keep an artificial plate in her mouth. That plate will get out of order. The filling Dr. Keely put into the tooth in the position God put it will be preferable, as every one will testify that it will stay longer than any artificial teeth that can be put there without repair. In the one case you have an artificial denture with rubber, a nonconductor of heat, acting upon the mucous membrane of the mouth, and when she would refresh herself with a draught of cold water, she does not get that pleasant sensation she would if she had her natural teeth. Away with your artificial teeth or throw them to the dogs. In the one case the teeth are there in the position God put them in—in the other as man put them in.

Dr. I. WILLIAMS thought there were, perhaps, but three cases in which he cheerfully and willingly extracted teeth. One was in extreme old age, where the processes are absorbed away and the teeth have become loose, causing irritation. Another was in the case of temporary teeth, where the permanent ones are making their appearance, and the third in cases where the teeth are very much crowded, and it is absolutely necessary to take out one on each side to give them room.

He would not be understood that he would not extract teeth in other cases. In such cases as had been referred to, where, perhaps, all the teeth in the mouth are decayed, and where the patient is absolutely too poor to give the time and attention to them, he might, in some such cases, with reluctance extract them, but with him, cases with such indications were very few. He was pleased with a remark he had seen in the REGISTER some time ago, with regard to saving even the roots of teeth. He had thought upon it much since that, and from what he had seen he was satisfied that even the roots of the teeth should be much valued. He had seen a root filled with gold twenty-five years ago, and it was still performing the part of a tooth well, even though it was but a root, and the processes had become firm and the root used as a tooth had become to look like a tooth. He was in favor of saving the roots and all the teeth that could be saved.

On motion of Dr. KEELY Dr. HARDY was invited to participate in the discussions on this subject.

Dr. HARDY said he would make a few remarks in reference to the extraction of the lateral incisors, because of crowded condition of the teeth. He believed he had some experience in the regulation of teeth, and he would differ from those who advocated the idea of extracting any of the incisor teeth, lateral or central. He believed Dr. Keely came nearer his idea of regulating teeth than any thing he had heard or seen for many years. He had not had a great deal of practice—only some twenty-five years. While he believed he knew something about regulating teeth, he believed Dentists generally are apt to neglect the regulation of teeth. He had often noticed in his practice in Cincinnati, where he had been for the last eighteen years, where they had extracted the lateral incisor to regulate the teeth. It was the simplest thing in the world to regulate the teeth, if a man knew what he was about. Dr. Hardy then proceeded to give his plans for regulating the teeth in certain cases.

Dr. Rehwinkle thought a great many remarks had been made on the subject, which might be summed up about as follows: The starting point would be that we would take it for granted that every body knows and appreciates the value of the natural teeth, and makes it his principle to save as many teeth as possible, and extract as few and as seldom as they possibly can, and then be a little more liberal in our ideas. He would not criticise too severely every individual case that did not meet our views, for if we could look at those cases they would, perhaps, present new features to us, upon a closer examination, so that we might find reasons for extracting teeth where we had supposed we would not. Where we can not, by argument or other means, bring our patients to the point to see what is best for them, and where they will not submit to the inconvenience of appliances, or the loss of time for a proper treatment, and if they are suffering severely, or annoyed by a malformation of the mouth or teeth, if we do the next best thing, we are doing our duty and all that can be asked. The subject of the extraction of teeth is a very broad one, and it is impossible to lay down rules to govern every case. Every man must be his own judge in each particular case, but before starting out every one ought to be well qualified and possess the necessary knowledge, and sufficient judgment to look a little ahead to the future in reference to the extraction of teeth.

He had reference now more particularly to the temporary teeth which it might be necessary to extract, on account of the condition of the mouth. It required considerable judgment and some experience to know how far nature will be capable of remedying a difficulty, if let alone, and when it is necessary to interfere. That ascertained, any practitioner who honestly and sincerely desires to do his duty will not have much trouble in getting along.

